# DNA methylation changes induced by long and short photoperiods in *Nasonia*

**DOI:** 10.1101/gr.196204.115

**Published:** 2016-02

**Authors:** Mirko Pegoraro, Akanksha Bafna, Nathaniel J. Davies, David M. Shuker, Eran Tauber

**Affiliations:** 1 Department of Genetics, University of Leicester, Leicester LE1 7RH, United Kingdom;; 2 School of Biology, University of St Andrews, St Andrews KY16 9TH, United Kingdom

## Abstract

Many organisms monitor the annual change in day length and use this information for the timing of their seasonal response. However, the molecular mechanisms underlying photoperiodic timing are largely unknown. The wasp *Nasonia vitripennis* is an emerging model organism that exhibits a strong photoperiodic response: Short autumnal days experienced by females lead to the induction of developmental arrest (diapause) in their progeny, allowing winter survival of the larvae. How female *Nasonia* control the developmental trajectory of their offspring is unclear. Here, we took advantage of the recent discovery that DNA methylation is pervasive in *Nasonia* and tested its role in photoperiodism. We used reduced representation bisulfite sequencing (RRBS) to profile DNA methylation in adult female wasps subjected to different photoperiods and identified substantial differential methylation at the single base level. We also show that knocking down *DNA methyltransferase 1a (Dnmt1a)*, *Dnmt3*, or blocking DNA methylation pharmacologically, largely disrupts the photoperiodic diapause response of the wasps. To our knowledge, this is the first example for a role of DNA methylation in insect photoperiodic timing.

Seasonal timing is a key survival process for many organisms, especially in temperate regions. In most cases, light is the primary cue, and the seasonal change in day length is monitored by a so-called “photoperiodic clock” (Bradshaw and Holzapfel 2010). Despite intensive study of the photoperiodic clock for the last 80 years at the phenomenological level, the “black box” approach has provided almost no information about the underlying molecular mechanisms (Saunders et al. 2004). This is in marked contrast to our level of understanding of the circadian clock that regulates daily rhythms, in which studies in various model organisms have led to the discovery of general principles and a detailed understanding of the underlying molecules, which are highly conserved across diverse phyla (Hogenesch and Ueda 2011).

*Nasonia* is a typical temperate-zone insect, in which short photoperiods experienced by females during autumn induce developmental arrest (diapause) in the progeny at the larval stage (Saunders 1965), an adaptation that allows the species to survive the winter. However, how the photoperiodic information is transferred from mothers to their progeny remains unknown, but other studies of the transgenerational transfer of phenotypes (Ho and Burggren 2010) suggest a possible role for epigenetic encoding (e.g., DNA methylation).

The recent sequencing of the *Nasonia* genome has revealed a comprehensive kit of DNA methylation machinery (Werren et al. 2010), including five DNA methyltransferase genes (*Dnmt1*-*3*). In a recent survey of methylation in *Nasonia* (Park et al. 2011), 18 genes were analyzed using bisulfite sequencing. On average, 30% of CpGs were methylated, indicating substantial methylation in the *Nasonia* genome, and as with other insects, this methylation was mainly found in gene bodies. The degree of methylation of the small subset of genes studied in *Nasonia* mirrored that seen for orthologous genes in other Hymenoptera (Zemach et al. 2010); and given the role of DNA methylation in phenotypic plasticity in honeybees (Kucharski et al. 2008), this process may well have an important role in regulating aspects of life history in *Nasonia* as well. Indeed, recent work exploring the RNAi silencing of *Dnmt1a* in *Nasonia* resulted in embryonic lethality, demonstrating a critical role of methylation in the control of development (Zwier et al. 2012).

The robust photoperiodic response of *Nasonia* provides us with the opportunity to test the role of DNA methylation in seasonal timing. Some evidence already alludes to this as a potential mechanism (Alvarado et al. 2014). For example, it has been shown that the timing of flowering in plants (induced by seasonal changes in day length) involves various epigenetic modifications, including DNA methylation (Yaish et al. 2011). Moreover, in mammals, DNA methylation of the type III deiodinase (*dio3*) gene has recently been shown to be critical for measuring photoperiod (Stevenson and Prendergast 2013).

Here, we test whether a similar encoding of environmental cues by DNA methylation underpins seasonal timing in insects. First, we provide a detailed genomic description of methylation in *Nasonia vitripennis*, which augments the recently published data sets (Wang et al. 2013; Beeler et al. 2014). Second, we identify differentially methylated sites in females associated with experience of long or short days. We predict that the major developmental decision of direct development versus diapause of offspring should involve genes in a number of pathways, some of which may respond to day length by increased methylation and some by decreased methylation. Third, we experimentally manipulate DNA methylation in females and observe how these manipulations influence offspring diapause. We use two independent techniques that influence genome-wide methylation in different ways: (1) RNA-interference knockdown of *Dnmt1a*–*c* and *Dnmt3*; and (2) pharmacological knockdown using the methylation inhibitor 5-aza-2′-deoxycytidine.

## Results

### The *Nasonia* methylome

To identify DNA methylation changes induced by photoperiod, we quantified DNA methylation in genomic DNA extracted from whole female *Nasonia vitripennis* wasps kept in either long- or short-day conditions. We used reduced representation bisulfite sequencing (RRBS) (Gu et al. 2011) to achieve single-nucleotide resolution of DNA methylation of CpG dinucleotides distributed throughout the *Nasonia* genome at high coverage.

After removal of low-quality reads, we obtained 27,255,357 reads (1.06 Gbp) from the long-day sample (LD) and 30,818,609 reads (1.25 Gbp) from the short-day sample (SD). The mapping efficiency of these reads to the *Nasonia* reference genome was 84.1% (LD) and 83.2% (SD). The average coverage depth of the reads was 92.6× for the short-day sample and 86.4× for the long-day sample. By the same metric, 4.53% (1,270,716) of CpG sites in the genome were covered by both samples at an average depth of 95.72× for the SD sample and 88.97× for the LD sample. The LD reads covered 1,304,087 CpG sites (4.65% of all genomic CpGs), whereas the SD reads covered 1,385,067 CpG (4.94%). We detected 1618 and 2018 methyl CpGs in the LD and SD samples, respectively, using a 1% false discovery rate (FDR) (see Methods), comprising 0.124% and 0.146% of the cytosines with sequence coverage that were analyzed.

**Figure 1.**
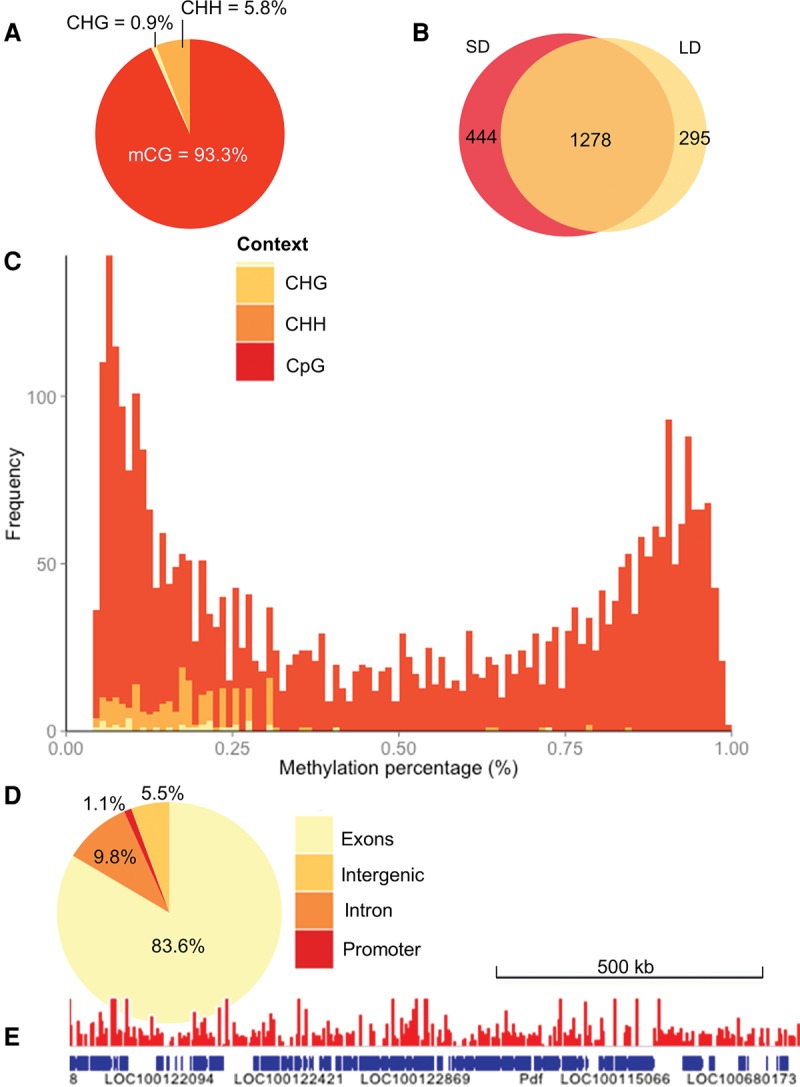
The *Nasonia* methylome. (*A*) Distribution of mCs identified in different sequence contexts (mCG, mCHG, and mCHH, where H = non-G). (*B*) Venn diagram showing that a substantial proportion of the mCGs are methylated under both long- and short-day conditions in contrast to other sequence contexts that show negligible overlap (see text), suggesting that non-CpG methylation represents largely experimental noise. (*C*) The methylation level of CpG sites (percentage of covering reads that are methylated) shows a bimodal distribution. This bimodality is not seen in CHG or CHH (shaded yellow and orange) methylation contexts. (*D*) Fraction of mCGs identified in exon, intron, promoters (2-kb upstream of genes), and intergenic regions. (*E*) A snapshot of the methylome (751-kb region) is depicted overlaid with the raw long-day methylation data (scaled to 0%–2% methylation).

Although most of the methylated cytosines were in a CpG context (Fig. 1A), a small fraction (<6.7%) of these were in non-CpG context (mCHG and mCHH, where H = C,T, or A) in both the LD and SD samples (Fig. 1A). CpG methylation shows a large amount of overlap between the two samples (Fig. 1B), whereas the other contexts show little (CHH; 1/125 sites), or no overlap (CHG), which may suggest that detected non-CpG methylation largely represents experimental noise (see Methods).

The methylation of CpG sites shows a bimodal distribution (Fig. 1C): CpGs seem to be either heavily methylated or lightly methylated (the methylation level of a cytosine is here defined as the percentage of reads that are methylated). Similar bimodality has been observed in both the honeybee and silk moth (Zemach et al. 2010), and methylation is negatively correlated with the CpG observed/expected ratio (a measure of CpG methylation), which is also bimodal in honeybees (Sarda et al. 2012) and *Nasonia* (Park et al. 2011). The majority (83.6%) of the methylated CpGs were located in exons, whereas only 9.8% were located in introns, 1.1% in promoter regions, and 5.5% in intergenic regions (Fig. 1D,E).

### Identification of differentially methylated genes

**Figure 2.**
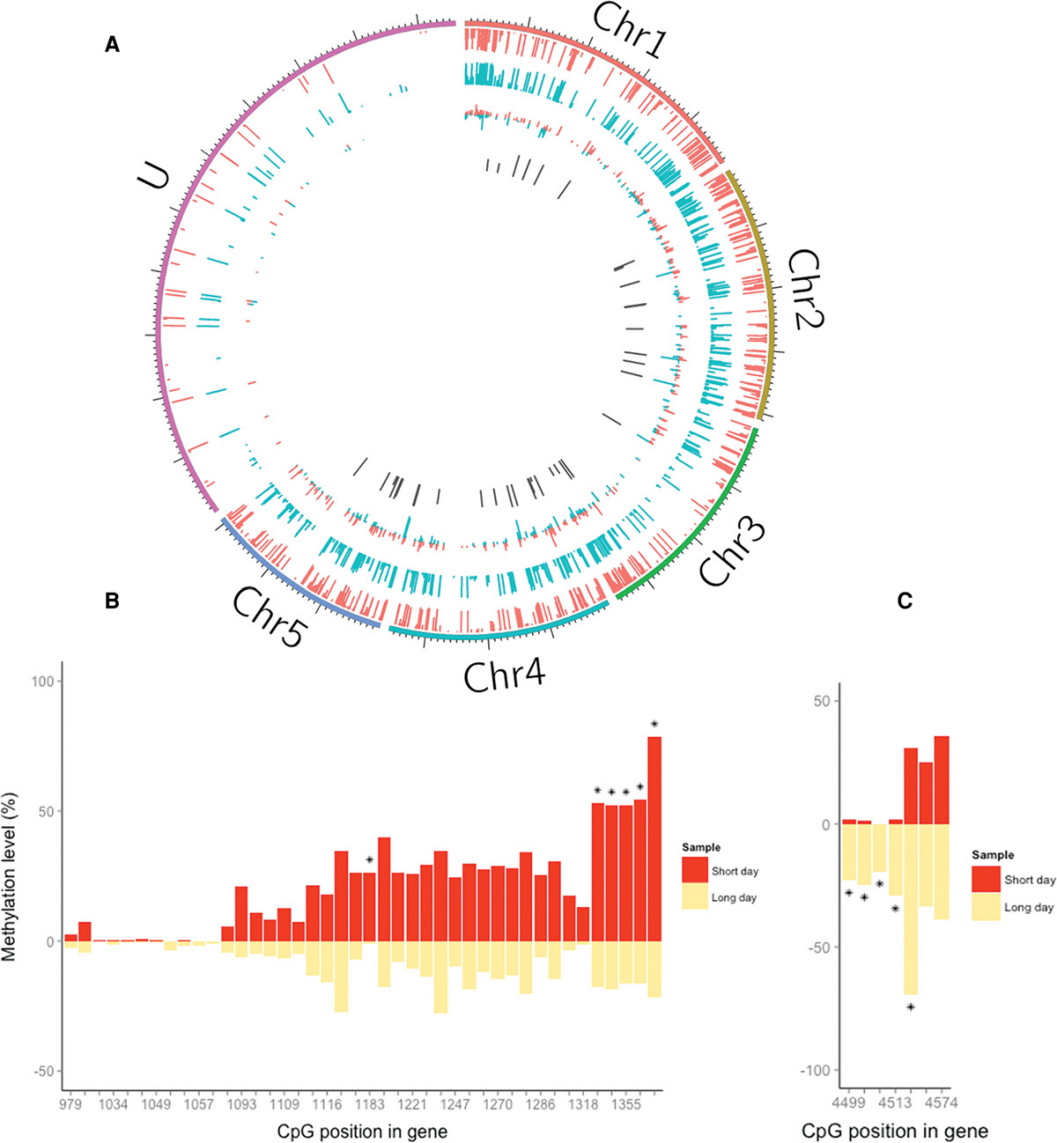
Differential DNA methylation associated with photoperiod in *Nasonia*. (*A*) The methylation is shown across the five chromosomes and the unplaced (U) scaffolds. From the *outer* to *inner* circle, the data tracks are short-day methylated CpGs (red), long-day methylated CpGs (blue), methylation difference (red/blue), and significantly differentially methylated sites (*q* < 0.05, dark gray). The lack of differential methylation in the U scaffolds is probably because they encompass substantially fewer genes. (*B*) Differential methylation in the *heix (*LOC100118618) gene. Of 42 CpG sites that were covered, six (*) were significantly more methylated in short day (*q* < 0.05). (*C*) Differential methylation in *msn* indicates increased methylation in long day: There are seven covered CpG sites on this gene, of which five (*) are methylated at *q* < 0.01.

Of the 12,055 genes with annotated mRNAs in the Nvit_2.0 build, 5972 (49.54%) had at least five covered CpG sites, and 5032 (41.74%) had at least 10 covered CpG sites. We used Fisher's exact tests to compare methylation in long and short photoperiods and identified 51 differentially methylated CpG sites (DMCs; Benjamini-Hochberg multiple testing, FDR < 0.05) (Benjamini and Hochberg 1995), which were mapped to 37 unique genes (Fig. 2A; Supplemental Table S1). Approximately half of the DMCs (23/51) showed reduced methylation in long day compared to short day (i.e., hypomethylation), whereas the other showed the opposite trend. Because the annotation of the *Nasonia* genome is still in its infancy, we were able to assign gene ontology (GO) terms only to 25 genes in our list. The terms assigned to the genes represented a wide range of functions, such as GTPase activity, protein binding, and transferase activity (acyl and glycosyl groups). A few of these genes harbored multiple DMCs, including *heixuedian* (*heix*), an ubiA prenyltransferase domain containing protein 1 gene. Of the 42 CpG sites in this gene that were covered by our library, six were significantly more methylated in the short-day sample (Fig. 2B). In contrast, the gene *misshapen (msn),* encoding a protein kinase, was represented by seven CpGs sites in our reads, five of which showed increased methylation in the long-day sample (at the FDR-corrected *q* < 0.01) (Fig. 2C). We have validated the differential methylation of specific CpG sites in the genes *WDR36*, (CG9799, WD repeat-containing protein 36, putative), LOC100117390 (CG11148, PERQ amino-acid rich with GYF domain-containing protein), and LOC100117821 (zinc finger protein 615) using a qPCR approach (Thomassin et al. 2004). In all three cases, we found a significant increase in methylation in long-day compared to short-day females, consistent with the RRBS analysis (three biological replicates, 2000 permutations, *P* < 0.0001) (Supplemental Fig. S1).

During bisulfite conversion, 5-hmC has been shown to behave like its precursor, 5-mC (Huang et al. 2010). We have therefore sought to test for the presence of 5hmC in our data. Recent studies in mammals have shown that 5hmC is made from a 5mC precursor, a reaction which is mediated by ten-eleven translocation (TET) proteins (Tahiliani et al. 2009; Ito et al. 2010). We have analyzed the *Nasonia* genome and identified a single putative Tet ortholog. Although the overall level of identity to human TET2 is rather low (0.25), the 925-bp Tet motif (Pfam ID: Tet_JBP) in the *Nasonia* gene results in a highly significant blast score (*E* = 4.9 × 10^−60^), suggesting it is a bona fide Tet ortholog. We carried out hydroxymethylated DNA immunoprecipitation (hMeDIP), using *Nasonia* gDNA that was spiked with mouse DNA (Thomson et al. 2013), and tested for the presence of three candidate genes in the enriched 5-hmC fractions by qPCR (Supplemental Fig. S2). The percent recovery (enriched/total input) of the genes *msn* and *heix* was <1%, similar to the mouse negative control, whereas the gene *Cact-2* was present at moderate level (1.1%–2.3%) compared to the positive control. Thus, functional 5-hMC might exist in *Nasonia* but is unlikely to play a significant role in the photoperiodic response. The complete survey of 5-hMC in *Nasonia* will be published elsewhere.

### Functional assays demonstrating a causative role of DNA methylation

**Figure 3.**
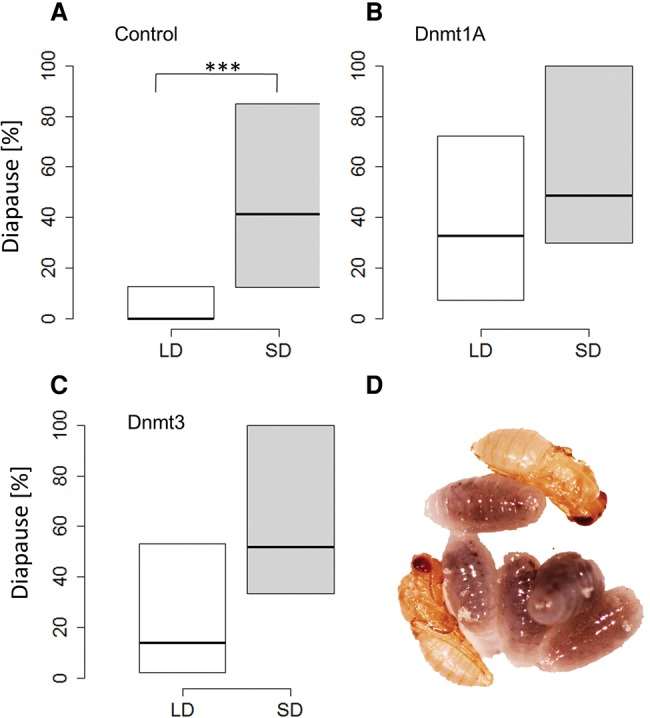
DNA methylation is required for photoperiodic-mediated diapause response. (*A*) The progeny of control females (GFP dsRNAi-injected) exhibit the normal photoperiodic response typical of *Nasonia*: low diapause incidence induced in long day (LD: 18-h light, white box) compared with short day (SD: 6-h light, gray box), as tested after 5 d at 18°C. (***) *P* < 0.01. In females injected with dsRNAi targeting *Dnmt1a* (*B*) and *Dnmt3* (*C*), the difference in diapause levels between long and short day is abolished (18–20 females in each group, progeny of each female collected from two hosts, average *n* = 24 larvae per female). The line *inside* each box represents the median; the *top* and the *bottom* represent the 75th and 25th percentiles, respectively. (*D*) *Nasonia* developing pupae (yellow) and diapausing larvae (dark).

To test the functional role of DNA methylation in mediating short-day-induced diapause, we knocked down *Dnmt1a-c* and *Dnmt3* by injecting double-stranded RNA (dsRNAi) of these genes into female pupae of the wild-type *N*. *vitripennis* strain AsymC. Control females were injected with dsRNAi targeting the green fluorescent protein (GFP). We verified the knockdowns by qPCR of mRNA extracted from the injected females (Supplemental Fig. S3). Although the complete knockdown of *Dnmt1a* is lethal (Zwier et al. 2012), our protocol induced a partial knockdown (∼60%) and resulted in the successful eclosion of adult females that were then tested for their photoperiodic responses. The diapause response of progeny of injected females is shown in Figure 3A. RNAi knockdown of *Dnmt1a* leads to females producing diapause offspring regardless of day length. Although control females exhibited the strong photoperiodic response typical of *Nasonia*, with increased diapause level in short days (*t* = 3.57, df = 28.6, *P* = 0.0012), the response of *Dnmt1a*-dsRNAi-injected females was similar in both long and short days (*t* = 1.45, df = 36, *P* = 0.15), with a substantial increase in diapause-induction in progeny of females maintained under long-day conditions. Silencing *Dnmt3* (Fig. 3B) resulted in a nonsignificant difference in diapause response, although this result is close to significance, suggesting that the effect of *Dnmt3* is much weaker or harder to resolve (*t* = 1.96, df = 36, *P* = 0.055). This effect of the dsRNAi was observed within 10 d of injection. For progeny laid 10–15 d after injection, diapause frequencies reverted to normal levels again (i.e., low diapause incidence in long days; data not shown). We have also attempted the knockdown of *Dnmt1b* and *Dnmt1c*, but the progeny of the injected wasps showed normal photoperiodic response (Supplemental Fig. S4).

We verified the effect of the RNAi knockdown on CpG methylation by using qPCR (MethyQuant), 9–10 d post injection (5 d after eclosion) We analyzed the status of highly methylated CpG sites in three different genes (Supplemental Fig. S4): *CG11148* (LOC100117390), *WDR36* (CG9799), and LOC100117821. Knockdown of *Dnmt1* resulted in a significant reduction of methylation (*P* < 0.05, calculated by permutation) in both the *WDR36* and LOC100117821 loci, whereas for *Dnmt3* knockdown, the level of methylation was reduced in the treated group (DNMTs-dsRNAi) compared to the control group (GFP-dsRNAi) in each of the tested genes (*P* < 0.05).

**Figure 4.**
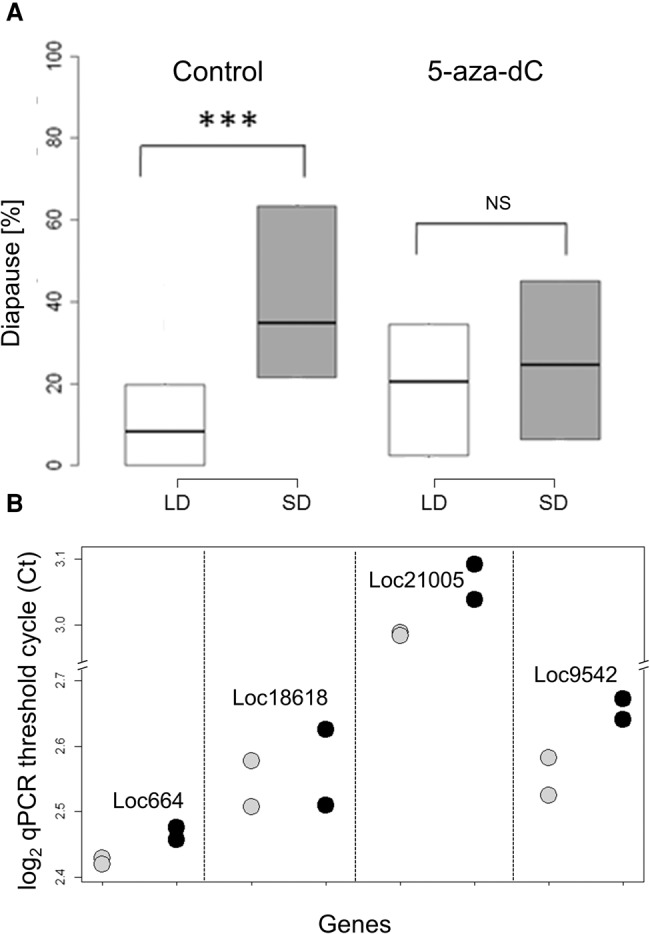
Blocking DNA methylation by 5-aza-dC. (*A*) Control females (*n* = 81) fed by sucrose solution exhibit the normal increased diapause in short day (SD, gray box) compared to long day (LD, light, white box), whereas females fed with 10 µM 5-aza-dC (*n* = 79) show aberrant photoperiodic response: (***) *P* < 0.01; (NS) not significant. (*B*) Reduction in the level of CpG methylation after drug treatment. The qPCR threshold cycle (log_2_ *C*_t_) values for control (gray circles) and drug-treated groups (black circles) is shown for four different loci. A clear increase (in three of the four loci) in *C*_t_ indicates reduced methylation (see text).

We also tested the impact of DNA methylation pharmacologically. We used the DNA methylation inhibitor 5-aza-2′-deoxycytidine (5-aza-dC), a potent inhibitor of DNA methylation (Christman 2002), and tested the female response to varying photoperiods. Although control females fed with sugar solution showed the normal photoperiodic response (again with a substantial difference between long- and short-day females; W = 338, *P* < 0.001) (Fig. 4A), wasps fed with 10 μM 5-aza-dC (a nonlethal dose), responded similarly to long and short days (W = 707, *P* = 0.47). Intriguingly, in this case, we observed a reduction of diapause in the progeny of wasps maintained in short day (median drop from 35% to 25%), accompanied by an increase in long-day progeny (8% to 20%). We verified the impact of the drug on DNA methylation of CpG sites in four different genes (Fig. 4B): LOC100121005 (*CG4049*, Helicase ARIP4), LOC100118618 (*heix*), LOC100679542 (*CG14322*, Zinc finger protein 106), and LOC100122664 (*CG5886*, Alpha-taxilin-like). A mixed effects model ANOVA (with the fixed effects Gene and Treatment, and replicate fitted as a random effect) revealed a significant reduction in methylation due to the drug (*F*_(1,8)_ = 9.45, *P* = 0.015). All genes exhibited the same response (interaction: *F*_(3,8)_ = 0.74, *P* = 0.56). We also tested the effect of 5-aza-dC on *msn* by cloning a 335-bp fragment of the gene using DNA from either treated or control females. Comparing the overall methylation in four CpG sites that were identified by RRBS revealed a significant reduction in the drug-treated group (*n* = 10 clones analyzed) compared to the control group (*n* = 9 clones): one methylated and 39 unmethylated sites compared to six methylated and 30 unmethylated sites (Fisher's exact test *P* = 0.048).

## Discussion

Here, we have shown that DNA methylation is associated with encoding the seasonal photoperiod in *Nasonia vitripennis*. Patterns of methylation across the genome change with photoperiod, and manipulation of the methylome via either the disruption of a key maintenance methyltransferase (*Dnmt1a*) or via a potent inhibitor of methylation (5-aza-dC) changes how female *Nasonia* respond to photoperiod. In addition, we have obtained a single-nucleotide resolution snapshot of the methylome of *Nasonia* that adds to the number of recently published insect methylomes, including the honeybee *Apis mellifera* (Lyko et al. 2010), the ants *Camponotus floridanus* and *Harpegnathos saltator* (Bonasio et al. 2012), the silk moth *Bombyx mori* (Xiang et al. 2010), and the locust *Schistocerca gregaria* (Falckenhayn et al. 2013). Moreover, the pattern of methylation in the wasp mirrors that of other insects, i.e., relatively low-density methylation, mostly mapped to gene bodies, particularly exons. Indeed, this seems to be an ancient property of eukaryotic genomes (Zemach et al. 2010), which is presumably involved in the regulation of alternative splicing (Lyko et al. 2010; Foret et al. 2012).

Profiling of DNA methylation by RRBS provides only a snapshot of the epigenome (Gu et al. 2011). It is therefore likely that the actual extent of differential methylation across the genome associated with the change in photoperiod is more substantial than that reported here (perhaps up to 20-fold higher, given our CpG coverage). It is also likely that our use of the whole body for methylation profiling hindered the detection of photoperiodic DMCs that may well be restricted to specific cell clusters in the brain or other tissues.

Of course, many of the DMCs we have identified may not necessarily be involved in the photoperiodic response (i.e., in inducing offspring diapause) because there are other physiological processes that might change with day length (for instance, involving changes in host-finding or resource allocation). Here, our goal has been to demonstrate that differential methylation with respect to photoperiod is present (although the link between a specific critical methylated locus and the photoperiodic circuit may be quite distant). Yet, some of the DMCs in our list may serve as candidates to follow. For instance, the gene *msn* has been shown to be involved in development in *Drosophila,* where it interacts with the JNK pathway; intriguingly, it is important for the neural development of photoreceptors (Treisman et al. 1997) and therefore may play a role in photoperiodic light reception.

Crucially, the disruption of the normal photoperiodic response by the knockdown of *Dnmt1a* demonstrates the causative role of DNA methylation in the photoperiodic response (Fig. 3). The observed abnormal increase in diapause incidence under long-day conditions following the RNAi manipulation suggests that under normal conditions, methylation of key photoresponse genes prevents diapause when days are long. However, the more pervasive pharmacological reduction of methylation by 5-aza-dC leads to diapause development under both long- and short-day conditions. In both cases, the photoperiodic response has been abolished, but not in quite the same way. This may be due to the fact that 5-aza-dC has a broad impact on DNA methylation (in contrast to *Dnmt1a* RNAi). As such, the differences in response in the two experiments may reflect multiple pathways and targets of DNA methylation with various effects on diapause, which are likely to be locus- and tissue-specific. Consistent with the fact that the change in day length is associated with both hyper- and hypomethylation (Fig. 2), the 5-aza-dC blocking is expected to impact both long- and short-day response.

In mammals, the function of DNMT1 is the maintenance of DNA methylation. If this function is conserved in *Nasonia*, then the effect of the knockdown would take place only in cells undergoing DNA replication. The 5-aza-dC treatment would impact both maintenance and de novo methylation, however, which might explain the differences in the observed phenotypes (Fig. 3). The de novo methylation might be accomplished by Dnmt3, whose activity would be carried out both in dividing and nondividing cells. Dnmt3 has been shown to be important for caste determination in honeybees (Kucharski et al. 2008) via the regulation of DNA methylation levels and alternative splicing (Li-Byarlay et al. 2013), and its role in the photoperiod response merits future investigation.

In contrast to *Dnmt1a*, the RNAi knockdown of *Dnamt1b-c* did not affect the photoperiodic response. This may be due to different expression patterns of the genes or different catalytic functions for Dnmt1b-c, which would be rather interesting given their sequence similarity. Similar functional differences were suggested by Zwier et al. (2012), in which inhibition of *Dnmt1a* (but not of *Dnmt1c*) during early embryogenesis led to developmental arrest and lethality. It is also possible that differences between the Dnmts are caused by their noncatalytic activity. Such noncatalytic regulation of gene expression has been demonstrated in the vertebrate *DNMT1* (Clements et al. 2012). The specific functional role of each of the *Nasonia* Dnmts again awaits further investigation.

Also noteworthy is that our methylome data show that photoperiodic transitions induce both up- and down-regulation of methylation, suggesting a complex regulation of methylation involving various methylating (and possibly demethylating) agents. Likewise, in honeybee caste determination, the methylation level in the brains of workers and queen shows both up- and down-regulation in a gene-specific manner (Lyko et al. 2010), which cannot be explained by merely the up-regulation of *Dnmts* in one of the castes.

In summary, our experiments clearly demonstrate a significant role of DNA methylation in photoperiodic timing. How these methylation changes underlie the maternal transfer of the photoperiodic response to the progeny remains to be determined. The fact that DNA methylation is involved in the photoperiodic clock echoes the role of epigenetic regulation in the circadian clock (Masri and Sassone-Corsi 2010; Ripperger and Merrow 2011). Indeed, most of those studies (carried out in mammals) underscored the role of histone modifications, which was also recently shown to be important for insect photoperiodism (Lu et al. 2013).

Whether photoperiodic timing is itself relying on the circadian clock is still a subject of debate (O'Brien et al. 2011), but some evidence suggests that this might be the case in *Nasonia* (Saunders 1974). If such a link between the two clocks does exist, it may involve light-induced epigenetic changes of Clock or Clock-controlled genes. Revealing the genes and cells that underlying the *Nasonia* circadian and photoperiodic clocks will soon allow a better understanding of the epigenetic mechanisms associated with these systems.

## Methods

### Wasp strains

We used the wild-type strain, AsymC, whose genome has been sequenced (Werren et al. 2010). To test for diapause, newly eclosed females were kept individually in glass vials containing two blowfly pupae (*Phaenicia cuprina*; hosts) at 18°C under various photoperiods (using LED illumination) for 10 d. Hosts were replaced twice, after 5 and 9 d, and kept in darkness at the same temperature for an additional 20 d. At this stage hosts, were dissected and diapause status of the progeny was determined (diapause larvae are easily identified through their morphology).

### RRBS library preparation

Newly eclosed females were maintained in either a long (18 h) or short (6 h) photoperiod. After 10 d, 2 h after lights on, genomic DNA was isolated from 10 females using the DNeasy kit (Qiagen). The RRBS libraries were prepared as previously described (Gu et al. 2011). Briefly, the gDNA (300 ng) was digested using MspI and the resulting CpG-terminated fragments were purified, and 3′ A-overhangs attached using Klenow fragment (3′-5′ exonuclease). The blunted end fragments were then ligated to methylated Illumina adapters (15 µM; Integrated DNA Technologies) that also included a 6-nt unique barcode at the 3′ end, allowing sequencing the samples in a single Illumina lane (see below). To obtain DNA fractions in the 40- to 200-bp range of MspI-digested products, the adapter-ligated fractions were excised from a 3% Nusieve gel. Bisulfite conversion was conducted using the EpiTect kit (Qiagen), following the manufacturer's instructions. The final libraries were generated by PCR amplification using PfuTurbo Cx Hotstart polymerase (2.5 U/µL; Agilent Technologies). The RRBS libraries were analyzed by an Agilent 2100 Bioanalyzer (Agilent Technologies).

### RRBS sequencing and data analysis

The libraries were sequenced using an Illumina HiSeq 2000 analyzer (BGI). The raw sequencing data was obtained through two Illumina sequencing runs on the same libraries, yielding 117,646,358 raw 50-bp reads in total. FastQC version 0.10.1 (http://www.bioinformatics.babraham.ac.uk/projects/fastqc) was used to provide data quality information at each step of the quality control process. The data were taken through several preprocessing steps before being aligned to the *Nasonia* genome (version Nvit_2.0) using Bismark v.0.7.7 (Krueger and Andrews 2011). These steps included de-multiplexing and barcode trimming, as well as trimming of low quality bases and Illumina adapter sequences from the 3′ end of the reads, using the Trim galore v0.2.5 package. Low quality reads (cutoff of 20 Phred score) were removed using FASTX-Toolkit (0.0.13; http://hannonlab.cshl.edu/fastx_toolkit/), which was also used to remove potential noise (i.e., reads that did not begin with YGG [where Y = C or T], as all RRBS reads are expected to begin with either CGG or TGG, in the nonconverted and converted cases, respectively). To facilitate downstream analysis, the resulting Bismark SAM output was sorted by chromosome and position using SAMtools version 0.1.18 (Li et al. 2009).

Once aligned to the genome, methylation sites were mapped by identifying C to T conversions produced by the bisulfite conversion. The error rate of methylation calling was estimated for each of the samples. Because the protocol uses unmethylated Cs for the filling in of the MspI restriction site, Cs at this site should always appear as Ts in the sequencing data. Any Cs that do appear in the sequencing data at this position are thus due to incomplete bisulfite conversion or T > C sequencing error. Only reads that contained the Illumina adapter and barcode at their 3′ end with an overlap of 11 bases or more were used to estimate the error because this was judged to be a good indication that the filled-in position could be located within these reads. The error estimate (E) due to incomplete bisulfite conversion and sequencing error is given by E = nCf/(nCf + nTf), where nCf is the number of Cs found at the filled-in position; and nTf is the number of Ts found at the filled-in position. The error estimates for each sample were 0.0068 (LD) and 0.00622 (SD).

To classify a cytosine as methylated (mC), the binomial distribution was used to calculate the probability of getting K successes (methylated Cs) in N trials (read depth) with probability E of a success (Lister et al. 2009). The *P*-values that had been generated in this way for each site were adjusted using the Benjamini-Hochberg method (Benjamini and Hochberg 1995), setting the FDR at 1%.

Differentially methylated Cs were identified using methylKit (Akalin et al. 2012) that uses the raw Bismark output data as an input. For each CpG, the proportions of methylated reads in the two photoperiods were compared using the Fisher's exact test, and the *P*-values were corrected to genomic-wide false discovery rate (FDR)-based *Q*-values by using the SLIM method (Wang et al. 2011). The methylated sites were mapped to genes using custom Perl scripts.

To test for 5-hydroxymethylation, we used the previously published hmeDIP protocol (Thomson et al. 2013). Briefly, genomic DNA (5 µg) was sonicated for 10 cycles, vacuum dried, and was spiked with mouse liver DNA (300 ng). Immunoprecipitation was carried with rabbit polyclonal antibody against hydroxymethylation (Active motif #39769) and purified (Qiagen). Enrichment of 5-hmC was tested using SYBR green qPCR in a 25-µL reaction volume using a two-step cycling program (15 min at 94°C and 45 min at 60°C) for 45 cycles. The target *Nasonia* genes were tested along mouse *Gapdh* and *Tex19.1* (negative and positive controls, respectively). Information about the tested genomic regions and primer sequences are in Supplemental Table S2.

### Validation of differential methylation by qPCR

We used the MethylQuant method (Thomassin et al. 2004) to validate specific differentially methylated CpGs in candidate genes. Following bisulfite conversion, the DNA was preamplified for 15 PCR cycles using nondiscriminatory (ND) primers (Supplemental Table S2). Three independent DNA samples were tested from long and short day for RNAi validation, and two independent replicates were used in testing the effect of the drug. The product was purified and used in qPCR (25 µL reaction volume) using discriminatory (D) primers harboring the methylation status-specific nucleotide at the 3′ end (Supplemental Table S2). The fluorescence data was analyzed using the qpcR library of the R statistical software (Ritz and Spiess 2008). The PCR efficiencies were averaged across replicates using the sliding window method, and ratios between the two conditions (long/short day) of a gene-of-interest were calculated using normalization against the nondiscriminatory qPCR data. Statistical significance for the ratios is calculated by a permutation approach (*n* = 2000) of randomly reallocated versus non-reallocated data (Ritz and Spiess 2008).

### Functional assays

The protocol for generation of *Dnmt1a*, *Dnmt3*, and GFP double-strand RNA and injection of pupae was previously described (Lynch and Desplan 2006). For the oligonucleotide sequences, see Supplemental Table S1. We verified that the dsRNAi fragments do not match the transcripts of any other genes (off-targets) using a custom-made off-target prediction tool, which we have made available online (http://WaspAtlas.com) (Davies and Tauber 2015). The tool dices the RNA sequence into all possible 19-mers, which are then matched against the *Nasonia* transcriptome (Nvit_2.0) and its complement.

Following injection, surviving females were collected and kept in different photoperiods as above. qPCR (SYBR green-based) was carried to determine transcript levels of the targeted gene, using the standard curve method. *Aequeorin* mRNA (a jellyfish photoreceptor) was spiked to the RNA samples and served as an exogenous reference (Gilsbach et al. 2006), and the *Nasonia RpL32* gene was used as an endogenous reference. Blocking DNA methylation by 5-aza-2-deoxycytidine was carried out by keeping newly eclosed female wasps individually at 25°C in LD 12:12 in the presence of 20% sucrose solution (200 µL) mixed with 10 µM of the drug (without a host). After 2 d, each female was given two hosts and transferred to 18°C at either a long- or short-day photoperiod. The hosts were collected after 5 d, and progeny were inspected after 20 d as above.

## Data access

All high-throughput sequencing data from this study have been submitted to the NCBI Gene Expression Omnibus (GEO; http://www.ncbi.nlm.nih.gov/geo/) under accession number GSE44869. The DNA methylation data can also be browsed at WaspAtlas (http://waspAtlas.com), our new *Nasonia* genomic database (Davies and Tauber 2015).

## Supplementary Material

Supplemental Material

## References

[PEGORAROGR196204C1] Akalin A, Kormaksson M, Li S, Garrett-Bakelman FE, Figueroa ME, Melnick A, Mason CE. 2012 methylKit: a comprehensive R package for the analysis of genome-wide DNA methylation profiles. Genome Biol 13: R87. 23034086 10.1186/gb-2012-13-10-r87 PMC3491415

[PEGORAROGR196204C2] Alvarado S, Fernald RD, Storey KB, Szyf M. 2014 The dynamic nature of DNA methylation: a role in response to social and seasonal variation. Integr Comp Biol 54: 68–76. 24813708 10.1093/icb/icu034 PMC4133575

[PEGORAROGR196204C3] Beeler SM, Wong GT, Zheng JM, Bush EC, Remnant EJ, Oldroyd BP, Drewell RA. 2014 Whole-genome DNA methylation profile of the jewel wasp (*Nasonia vitripennis*). G3 (Bethesda) 4: 383–388. 24381191 10.1534/g3.113.008953 PMC3962478

[PEGORAROGR196204C4] Benjamini Y, Hochberg Y. 1995 Controlling the false discovery rate: a practical and powerful approach to multiple testing. J R Statist Soc B 57: 289–300.

[PEGORAROGR196204C5] Bonasio R, Li Q, Lian J, Mutti NS, Jin L, Zhao H, Zhang P, Wen P, Xiang H, Ding Y, 2012 Genome-wide and caste-specific DNA methylomes of the ants *Camponotus floridanus* and *Harpegnathos saltator*. Curr Biol 22: 1755–1764. 22885060 10.1016/j.cub.2012.07.042 PMC3498763

[PEGORAROGR196204C6] Bradshaw WE, Holzapfel CM. 2010 Light, time, and the physiology of biotic response to rapid climate change in animals. Annu Rev Physiol 72: 147–166. 20148671 10.1146/annurev-physiol-021909-135837

[PEGORAROGR196204C7] Christman JK. 2002 5-Azacytidine and 5-aza-2′-deoxycytidine as inhibitors of DNA methylation: mechanistic studies and their implications for cancer therapy. Oncogene 21: 5483–5495. 12154409 10.1038/sj.onc.1205699

[PEGORAROGR196204C8] Clements EG, Mohammad HP, Leadem BR, Easwaran H, Cai Y, Van Neste L, Baylin SB. 2012 DNMT1 modulates gene expression without its catalytic activity partially through its interactions with histone-modifying enzymes. Nucleic Acids Res 40: 4334–4346. 22278882 10.1093/nar/gks031 PMC3378872

[PEGORAROGR196204C9] Davies NJ, Tauber E. 2015 WaspAtlas: a *Nasonia vitripennis* gene database and analysis platform. Database (Oxford) 2015: bav103. 26452372 10.1093/database/bav103 PMC4599445

[PEGORAROGR196204C10] Falckenhayn C, Boerjan B, Raddatz G, Frohme M, Schoofs L, Lyko F. 2013 Characterization of genome methylation patterns in the desert locust *Schistocerca gregaria*. J Exp Biol 216: 1423–1429. 23264491 10.1242/jeb.080754

[PEGORAROGR196204C11] Foret S, Kucharski R, Pellegrini M, Feng S, Jacobsen SE, Robinson GE, Maleszka R. 2012 DNA methylation dynamics, metabolic fluxes, gene splicing, and alternative phenotypes in honey bees. Proc Natl Acad Sci 109: 4968–4973. 22416128 10.1073/pnas.1202392109 PMC3324026

[PEGORAROGR196204C12] Gilsbach R, Kouta M, Bonisch H, Brüss M. 2006 Comparison of in vitro and in vivo reference genes for internal standardization of real-time PCR data. Biotechniques 40: 173–177. 16526406 10.2144/000112052

[PEGORAROGR196204C13] Gu H, Smith ZD, Bock C, Boyle P, Gnirke A, Meissner A. 2011 Preparation of reduced representation bisulfite sequencing libraries for genome-scale DNA methylation profiling. Nat Protoc 6: 468–481. 21412275 10.1038/nprot.2010.190

[PEGORAROGR196204C14] Ho DH, Burggren WW. 2010 Epigenetics and transgenerational transfer: a physiological perspective. J Exp Biol 213: 3–16. 20008356 10.1242/jeb.019752

[PEGORAROGR196204C15] Hogenesch JB, Ueda HR. 2011 Understanding systems-level properties: timely stories from the study of clocks. Nat Rev Genet 12: 407–416. 21556016 10.1038/nrg2972

[PEGORAROGR196204C16] Huang Y, Pastor WA, Shen Y, Tahiliani M, Liu DR, Rao A. 2010 The behaviour of 5-hydroxymethylcytosine in bisulfite sequencing. PLoS One 5: e8888. 20126651 10.1371/journal.pone.0008888 PMC2811190

[PEGORAROGR196204C17] Ito S, D'Alessio AC, Taranova OV, Hong K, Sowers LC, Zhang Y. 2010 Role of Tet proteins in 5mC to 5hmC conversion, ES-cell self-renewal and inner cell mass specification. Nature 466: 1129–1133. 20639862 10.1038/nature09303 PMC3491567

[PEGORAROGR196204C18] Krueger F, Andrews SR. 2011 Bismark: a flexible aligner and methylation caller for Bisulfite-Seq applications. Bioinformatics 27: 1571–1572. 21493656 10.1093/bioinformatics/btr167 PMC3102221

[PEGORAROGR196204C19] Kucharski R, Maleszka J, Foret S, Maleszka R. 2008 Nutritional control of reproductive status in honeybees via DNA methylation. Science 319: 1827–1830. 18339900 10.1126/science.1153069

[PEGORAROGR196204C20] Li H, Handsaker B, Wysoker A, Fennell T, Ruan J, Homer N, Marth G, Abecasis G, Durbin R, 1000 Genome Project Data Processing Subgroup. 2009 The Sequence Alignment/Map format and SAMtools. Bioinformatics 25: 2078–2079. 19505943 10.1093/bioinformatics/btp352 PMC2723002

[PEGORAROGR196204C21] Li-Byarlay H, Li Y, Stroud H, Feng S, Newman TC, Kaneda M, Hou KK, Worley KC, Elsik CG, Wickline SA, 2013 RNA interference knockdown of DNA methyl-transferase 3 affects gene alternative splicing in the honey bee. Proc Natl Acad Sci 110: 12750–12755. 23852726 10.1073/pnas.1310735110 PMC3732956

[PEGORAROGR196204C22] Lister R, Pelizzola M, Dowen RH, Hawkins RD, Hon G, Tonti-Filippini J, Nery JR, Lee L, Ye Z, Ngo QM, 2009 Human DNA methylomes at base resolution show widespread epigenomic differences. Nature 462: 315–322. 19829295 10.1038/nature08514 PMC2857523

[PEGORAROGR196204C23] Lu YX, Denlinger DL, Xu WH. 2013 Polycomb repressive complex 2 (PRC2) protein ESC regulates insect developmental timing by mediating H3K27me3 and activating prothoracicotropic hormone gene expression. J Biol Chem 288: 23554–23564. 23814061 10.1074/jbc.M113.482497 PMC3949329

[PEGORAROGR196204C24] Lyko F, Foret S, Kucharski R, Wolf S, Falckenhayn C, Maleszka R. 2010 The honey bee epigenomes: differential methylation of brain DNA in queens and workers. PLoS Biol 8: e1000506. 21072239 10.1371/journal.pbio.1000506 PMC2970541

[PEGORAROGR196204C25] Lynch JA, Desplan C. 2006 A method for parental RNA interference in the wasp *Nasonia vitripennis*. Nat Protoc 1: 486–494. 17406271 10.1038/nprot.2006.70

[PEGORAROGR196204C26] Masri S, Sassone-Corsi P. 2010 Plasticity and specificity of the circadian epigenome. Nat Neurosci 13: 1324–1329. 20975756 10.1038/nn.2668 PMC4071955

[PEGORAROGR196204C27] O'Brien C, Bradshaw WE, Holzapfel CM. 2011 Testing for causality in covarying traits: genes and latitude in a molecular world. Mol Ecol 20: 2471–2476. 21595769 10.1111/j.1365-294X.2011.05133.x PMC3362484

[PEGORAROGR196204C28] Park J, Peng Z, Zeng J, Elango N, Park T, Wheeler D, Werren JH, Yi SV. 2011 Comparative analyses of DNA methylation and sequence evolution using *Nasonia* genomes. Mol Biol Evol 28: 3345–3354. 21693438 10.1093/molbev/msr168 PMC3215512

[PEGORAROGR196204C29] Ripperger JA, Merrow M. 2011 Perfect timing: epigenetic regulation of the circadian clock. FEBS Lett 585: 1406–1411. 21536041 10.1016/j.febslet.2011.04.047

[PEGORAROGR196204C30] Ritz C, Spiess AN. 2008 *qpcR*: an R package for sigmoidal model selection in quantitative real-time polymerase chain reaction analysis. Bioinformatics 24: 1549–1551. 18482995 10.1093/bioinformatics/btn227

[PEGORAROGR196204C31] Sarda S, Zeng J, Hunt BG, Yi SV. 2012 The evolution of invertebrate gene body methylation. Mol Biol Evol 29: 1907–1916. 22328716 10.1093/molbev/mss062

[PEGORAROGR196204C32] Saunders DS. 1965 Larval diapause of maternal origin: induction of diapause in *Nasonia vitripennis* (Walk.) (Hymenoptera: Pteromalidae). J Exp Biol 42: 495–508.

[PEGORAROGR196204C33] Saunders D. 1974 Evidence for ‘dawn’ and ‘dusk’ oscillators in the *Nasonia* photoperiodic clock. J Insect Physiol 20: 77–88.

[PEGORAROGR196204C34] Saunders DS, Lewis RD, Warman GR. 2004 Photoperiodic induction of diapause: opening the black box. Physiol Entomol 29: 1–15.

[PEGORAROGR196204C35] Stevenson TJ, Prendergast BJ. 2013 Reversible DNA methylation regulates seasonal photoperiodic time measurement. Proc Natl Acad Sci 110: 16651–16656. 24067648 10.1073/pnas.1310643110 PMC3799317

[PEGORAROGR196204C36] Tahiliani M, Koh KP, Shen Y, Pastor WA, Bandukwala H, Brudno Y, Agarwal S, Iyer LM, Liu DR, Aravind L, 2009 Conversion of 5-methylcytosine to 5-hydroxymethylcytosine in mammalian DNA by MLL partner TET1. Science 324: 930–935. 19372391 10.1126/science.1170116 PMC2715015

[PEGORAROGR196204C37] Thomassin H, Kress C, Grange T. 2004 MethylQuant: a sensitive method for quantifying methylation of specific cytosines within the genome. Nucleic Acids Res 32: e168. 15576675 10.1093/nar/gnh166 PMC535695

[PEGORAROGR196204C38] Thomson JP, Hunter JM, Nestor CE, Dunican DS, Terranova R, Moggs JG, Meehan RR. 2013 Comparative analysis of affinity-based 5-hydroxymethylation enrichment techniques. Nucleic Acids Res 41: e206. 24214958 10.1093/nar/gkt1080 PMC3905904

[PEGORAROGR196204C39] Treisman JE, Ito N, Rubin GM. 1997 *misshapen* encodes a protein kinase involved in cell shape control in *Drosophila*. Gene 186: 119–125. 9047354 10.1016/s0378-1119(96)00694-4

[PEGORAROGR196204C40] Wang HQ, Tuominen LK, Tsai CJ. 2011 SLIM: a sliding linear model for estimating the proportion of true null hypotheses in data sets with dependence structures. Bioinformatics 27: 225–231. 21098430 10.1093/bioinformatics/btq650

[PEGORAROGR196204C41] Wang X, Wheeler D, Avery A, Rago A, Choi JH, Colbourne JK, Clark AG, Werren JH. 2013 Function and evolution of DNA methylation in *Nasonia vitripennis*. PLoS Genet 9: e1003872. 24130511 10.1371/journal.pgen.1003872 PMC3794928

[PEGORAROGR196204C42] Werren JH, Richards S, Desjardins CA, Niehuis O, Gadau J, Colbourne JK, Nasonia Genome Working Group, Werren JH, Richards S, Desjardins CA, 2010 Functional and evolutionary insights from the genomes of three parasitoid *Nasonia* species. Science 327: 343–348. 20075255 10.1126/science.1178028 PMC2849982

[PEGORAROGR196204C43] Xiang H, Zhu J, Chen Q, Dai F, Li X, Li M, Zhang H, Zhang G, Li D, Dong Y, 2010 Single base–resolution methylome of the silkworm reveals a sparse epigenomic map. Nat Biotechnol 28: 516–520. 20436463 10.1038/nbt.1626

[PEGORAROGR196204C44] Yaish MW, Colasanti J, Rothstein SJ. 2011 The role of epigenetic processes in controlling flowering time in plants exposed to stress. J Exp Bot 62: 3727–3735. 21633082 10.1093/jxb/err177

[PEGORAROGR196204C45] Zemach A, McDaniel IE, Silva P, Zilberman D. 2010 Genome-wide evolutionary analysis of eukaryotic DNA methylation. Science 328: 916–919. 20395474 10.1126/science.1186366

[PEGORAROGR196204C46] Zwier MV, Verhulst EC, Zwahlen RD, Beukeboom LW, van de Zande L. 2012 DNA methylation plays a crucial role during early *Nasonia* development. Insect Mol Biol 21: 129–138. 22122805 10.1111/j.1365-2583.2011.01121.x

