# Supplementary material for: DNA methylation changes induced by long and short photoperiods in Nasonia
Source: Genome Res. 2016 Feb;26(2):203–10. doi: 10.1101/gr.196204.115 (PMC4728373; doi:10.1101/gr.196204.115)
Supplement: Supplemental Material [file supp_26_2_203__index.html]

DNA methylation changes induced by long and short photoperiods in Nasonia — DNA methylation changes induced by long and short photoperiods in Nasonia — Supplemental Material 

# DNA methylation changes induced by long and short photoperiods in *Nasonia*

## Supplemental Material

**Files in this Data Supplement:**

- Supplemental Material.pdf
