# Supplementary material for: DNA methylation changes induced by long and short photoperiods in Nasonia
Source: Genome Res. 2016 Feb;26(2):203–10. doi: 10.1101/gr.196204.115 (PMC4728373; doi:10.1101/gr.196204.115)

The seasonal epigenome: Snapshots of DNA methylation changes between long and short photoperiods in *Nasonia*

Mirko Pegoraro, Akanksha Bafna, Nathaniel J. Davies, David M. Shuker, and Eran Tauber

Supplementary Material

Table S1. Top differentially methylated CpG sites (q < 0.05). For each site, the difference in methylation level in LD relative to SD (5mC Δ) and Fisher's exact test FDR (q-value) are shown.

Gene name	Gene ID	5mC Δ	q-value	NCBI full name
LOC100123268	NV11549	-77.59	5.30E-007	probable tRNA threonylcarbamoyladenosine biosynthesis protein osgep-like
LOC100118618	NV15033	-56.87	5.09E-013	ubiA prenyltransferase domain-containing protein 1 homolog
LOC100114611	NV17739	-43.88	1.15E-008	uncharacterized glycosyltransferase AER61-like
LOC100122036	NV13848	-42.88	1.23E-004	carboxy-terminal domain RNA polymerase II polypeptide A small phosphatase 1-like
LOC100114164	NV16579	-38.04	4.66E-007	endoplasmin-like
LOC100118618	NV15033	-37.87	8.71E-005	ubiA prenyltransferase domain-containing protein 1 homolog
Lin		-37.21	6.20E-003	lines
LOC100118618	NV15033	-35.85	5.06E-004	ubiA prenyltransferase domain-containing protein 1 homolog
LOC100122046	NV12262	-35.64	6.36E-005	chromodomain-helicase-DNA-binding protein Mi-2 homolog
LOC100118618	NV15033	-35.49	6.64E-004	ubiA prenyltransferase domain-containing protein 1 homolog
LOC100118618	NV15033	-33.79	3.11E-003	ubiA prenyltransferase domain-containing protein 1 homolog
Atp5b	NV12146	-33.12	1.01E-002	ATP synthase, H+ transporting, mitochondrial F1 complex, beta polypeptide
LOC100120295	NV16720	-31.33	1.90E-002	uncharacterized LOC100120295
LOC100122200	NV13851	-31.29	4.61E-003	frizzled-7-like
LOC100122869	NV12317	-30.35	4.22E-002	carnitine O-palmitoyltransferase 2, mitochondrial-like
LOC100117948	NV14276	-29.45	3.87E-002	zinc finger protein Helios-like
LOC100114164	NV16579	-27.28	1.60E-004	endoplasmin-like
LOC100118618	NV15033	-25.32	2.02E-004	ubiA prenyltransferase domain-containing protein 1 homolog
LOC100123019	NV11527	-23.34	2.27E-002	N-acetyltransferase 15-like
LOC100114300	NV18104	-22.44	5.62E-005	eukaryotic translation initiation factor 4H-like
LOC100115546	NV15853	-16.67	4.22E-002	inositol hexakisphosphate and diphosphoinositol-pentakisphosphate kinase-like
LOC100115582	NV15403	-12.76	1.90E-002	girdin-like
LOC100114300	NV18104	-12.05	1.06E-002	eukaryotic translation initiation factor 4H-like
LOC100113804	NV13108	16.88	1.69E-002	60S ribosome subunit biogenesis protein NIP7 homolog
msn	NV18625	19.27	2.92E-004	misshapen
LOC100119027	NV15549	20.80	4.22E-002	T-complex protein 1 subunit beta-like
msn	NV18625	20.87	5.67E-003	misshapen
msn	NV18625	23.39	1.48E-004	misshapen
msn	NV18625	27.29	1.28E-005	misshapen
LOC100116941	NV13337	27.52	4.22E-002	HBS1-like protein-like
LOC100122518	NV14674	28.05	1.90E-003	uncharacterized LOC100122518

Gene name	Gene ID	5mC Δ	q-value	NCBI full name
LOC100119184	NV12166	31.71	1.43E-006	uncharacterized LOC100119184
msn	NV18625	38.63	1.02E-005	misshapen
Cact2	NV11366	39.24	1.09E-009	NF-kappa-B inhibitor cactus 2
LOC100114102	NV17553	61.14	3.68E-003	uncharacterized LOC100114102
Brm	NV11766	72.94	6.19E-003	ATP-dependent helicase brahma
LOC100118729	NV14558	75.71	3.35E-007	nodal modulator 1-like
LOC100118687	NV14557	75.71	3.35E-007	uncharacterized LOC100118687
LOC100115227	NV16159	79.82	1.01E-008	ubiquitin C-like
LOC100121209	NV13544	84.41	1.96E-006	ubiquitin carboxyl-terminal hydrolase 36-like
LOC100679730		89.35	4.60E-011	protein cappuccino homolog
LOC100117025	NV15224	89.35	4.60E-011	dolichyl-diphosphooligosaccharide--protein glycosyltransferase subunit STT3B-like
mor	NV13586	89.87	1.15E-004	moira
WDR36		94.74	1.15E-008	WD repeat domain 36
LOC100680213		97.73	8.74E-008	uncharacterized LOC100680213
LOC100119692	NV18435	97.73	8.74E-008	WD repeat-containing protein 20-like
LOC100117821	NV50484	99.26	3.00E-030	zinc finger protein 132-like
LOC100117390	NV18148	100.00	1.56E-010	PERQ amino acid-rich with GYF domain-containing protein 2-like

Table S2. Sequence and coordinates of oligonucleotides used in qPCR for RRBS validation, and 5hmC analysis.

Gene (primer used)	Forward primer (5'-3')	Reverse primer (5'-3')	Gene ID (NCBI)	Coordinates	Amplicon size (bp)
MSN	CATTTATCCGCGATCAACCT	AGCTGGCTCATCATCTTCGT	341864961	17786041-17786183	143
UbiA-1	GACCGATATCCGTGCTGTTC	TCTCGAGGTCCCTGGTATTG	341864959	c29619956-29620087	132
Cact2	GGACAAACAGCAGGTGGATT	GCAAGCCTTAGCAGGTTCAG	341864958	c 2065624-2065771	148
LOC100117821			341864960	c7593895-7593681	215
Pre-amplification	TTGGTTATGTTATATGGTTATTTAG	TATCTACTAACAACTCTCATTCATC			
		TCTAATCCTAATTATTATTAACC		c7593883-7593782	102
Discriminative	TATGGTTATTTAG/ideoxyI/GGTTT+C				
Non-discriminative	TATGGTTATTTAG/ideoxyI/GGTTT				
WDR36			341864959		
Pre-amplification	TAATTTGGGGATTTTTTTGTGG	TCAATATTCAACAAATTCAACCAAC		c 30423392-30423117	276
				c 30423277-30423148	130
Discriminative	GTTATTTTAGATTTTTTAGATAGTA+C				
Non-discriminative	GTTATTTTAGATTTTTTAGATAGTA	TAACCAAAACAAACATAATAAC			
LOC100117390					
Pre-amplification	TTATTTGTAAAAATGTATTTTTTTT	ATTATAACACCAATTAATTCTACTC	341864961	14674461-14674739	279
				14674567-14674679	
Discriminative	GTTTG/ideoxyI/GAATTAGTTGGATT+C				
Non-discriminative	GTTTG/ideoxyI/GAATTAGTTGGAT	ATAACTTATTAATAACACTTACCC			113

Fig. S1. **Validation of differential methylation by qPCR.** Differential methylation of specific cytosine in three genes was tested by MethylQuant, a qPCR approach (Thomassin,H. 2004). Boxplot showing the median and 95% confidence limits of the sample ratios calculated by permutation using the qpcR library of the R software. All loci show a significant increase in methylation in long day, in accordance with the RRBS analysis.

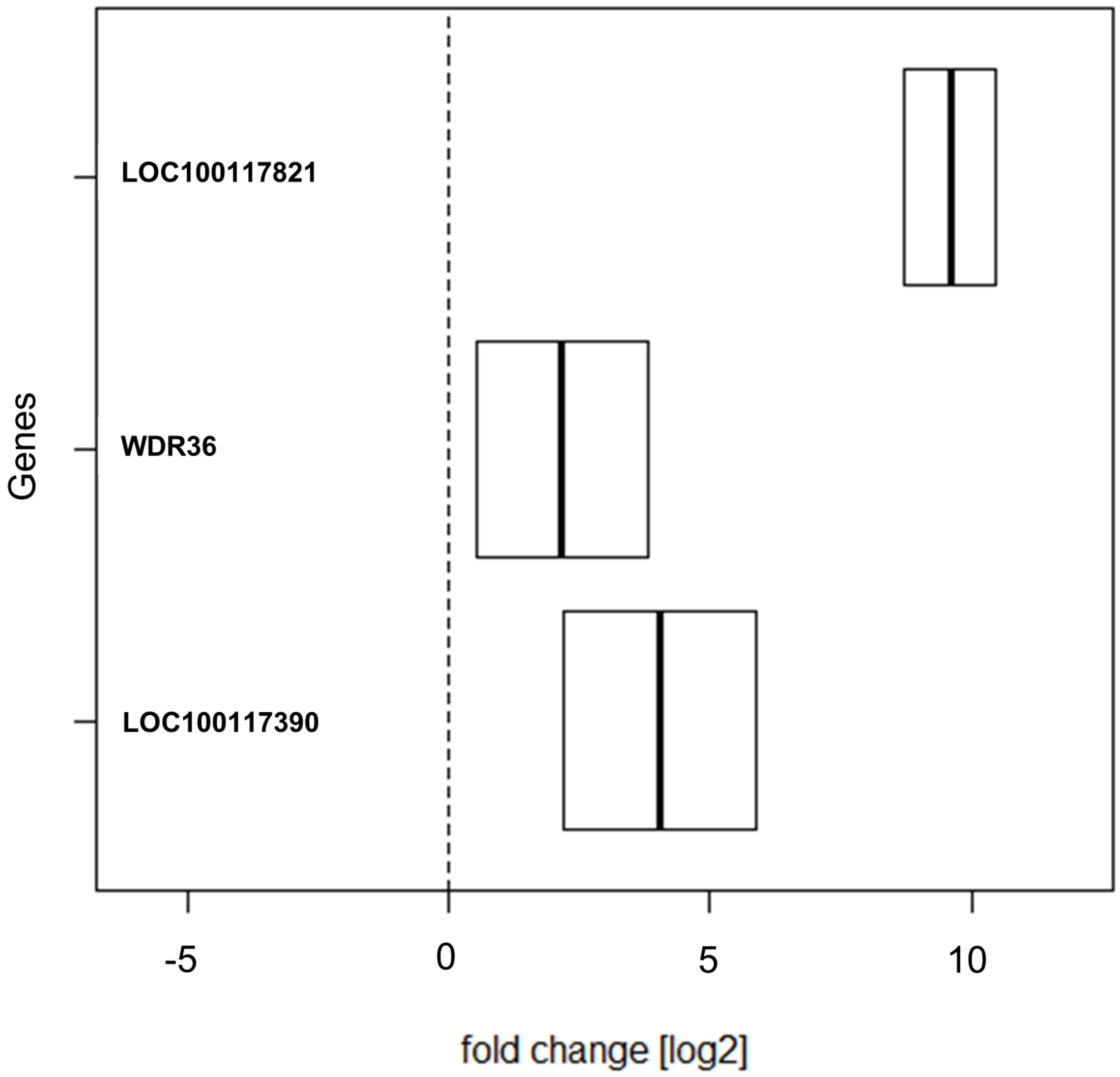

Fig. S2. **Levels of 5-hydroxymethylation in candidate genes.** *Nasonia* genomic DNA (spiked with mouse DNA) was analysed by hMeDIP using antibodies against 5hmC. Immunoprecipitated DNA was amplified by gene-specific q-PCR. The mouse gene *GAPDH* and *Tex19* serves as negative and positive controls respectively. Three independent experiments are shown.

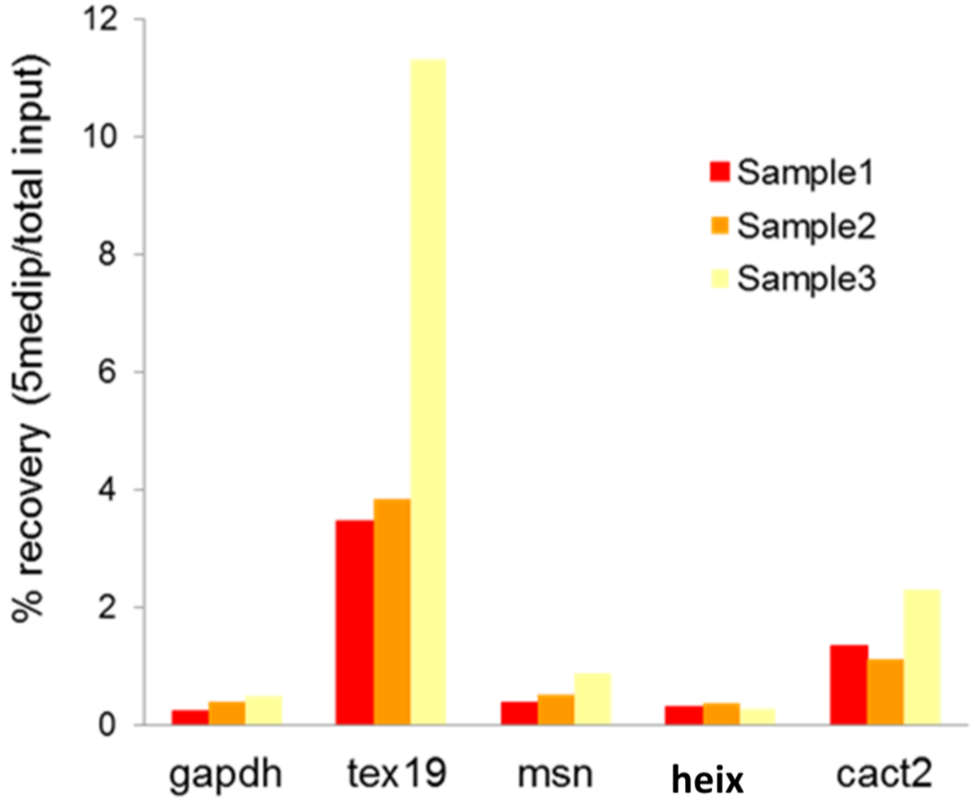

Fig. S3. **Validation of knock-down of *DNMTs*.** The normalized expression level of **A**. *Dnmt1a* **B**. *Dnmt1b* **C**. *Dnmt1c and* **D**. *Dnmt3* were compared in the treated group (n=30; white boxplots) to the control group (n=30, grey box plots). The line inside each box represents the median, the top and the bottom the 75 and 25th percentiles respectively, error bars range from minimum to maximum (excluding outliers). Significance codes as per TukeyHSD (ANOVA): 0 '***' 0.001 '**' 0.01 '*' 0.05 '.' 0.1 ' ' 1

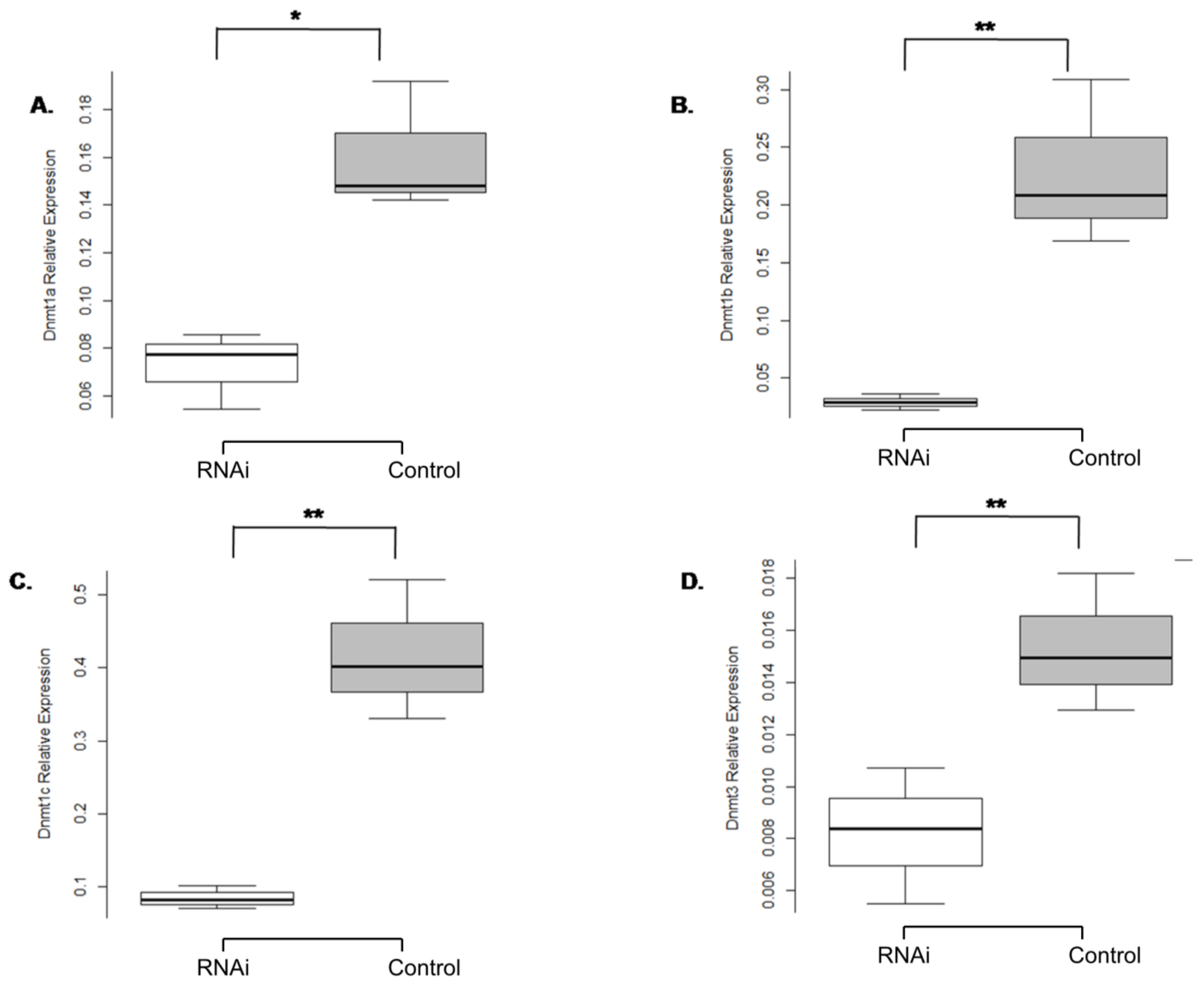

Fig. S4. **Level of Diapause after *DNMTs* knock-down**. The progeny of females injected with *Dnmt1b* and *Dnmt1c* dsRNAi was tested after 5 days at 18°C. In both cases normal photoperiodic response typical of *Nasonia* was observed: low diapause incidence induced in long day (LD, 18 hr light, white box) compared with short day (SD, 6 hr light, grey box) is seen. 18-20 females in each group, progeny of each female collected from two hosts, average n=24 larvae per female. The line inside each box represents the median, the top and the bottom the 75 and 25th percentiles respectively, error bars range from minimum to maximum (excluding outliers).Significance Codes as per TukeyHSD (ANOVA): 0 ‘***’ 0.001 ‘**’ 0.01 ‘*’ 0.05 ‘.’ 0.1‘ ’ 1.

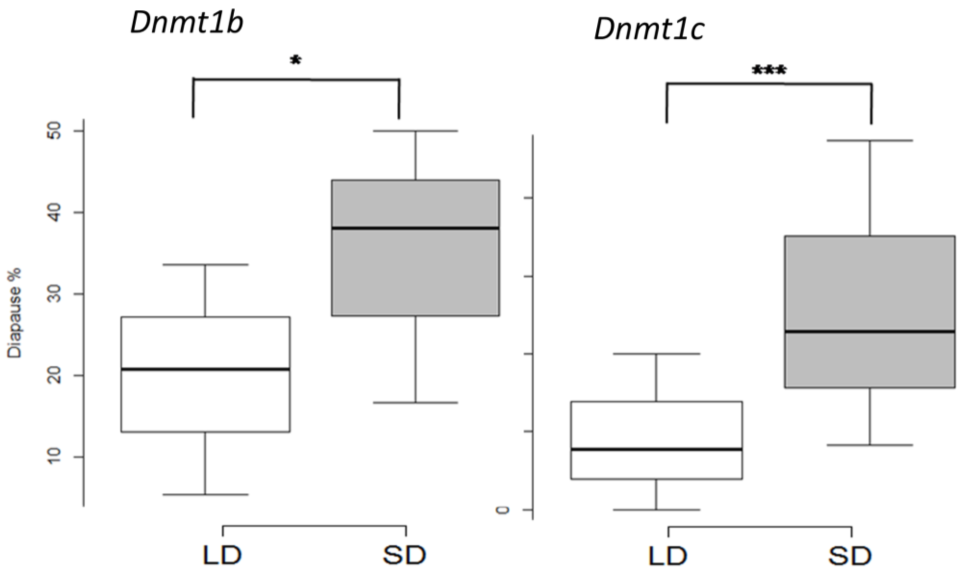

Fig. S5. Reduced methylation after knockdown of Dnmt1a (bottom) and Dnmt3 (top). Boxplots depict the distribution of the replicates and the error estimated through permutation using the qpcR library of the R software. The dotted line describes the value for which the ratio is one (i.e. no difference in methylation). Differential methylation of specific cytosine in each genes was tested by MethylQuant, a qPCR approach (Thomassin. 2004).

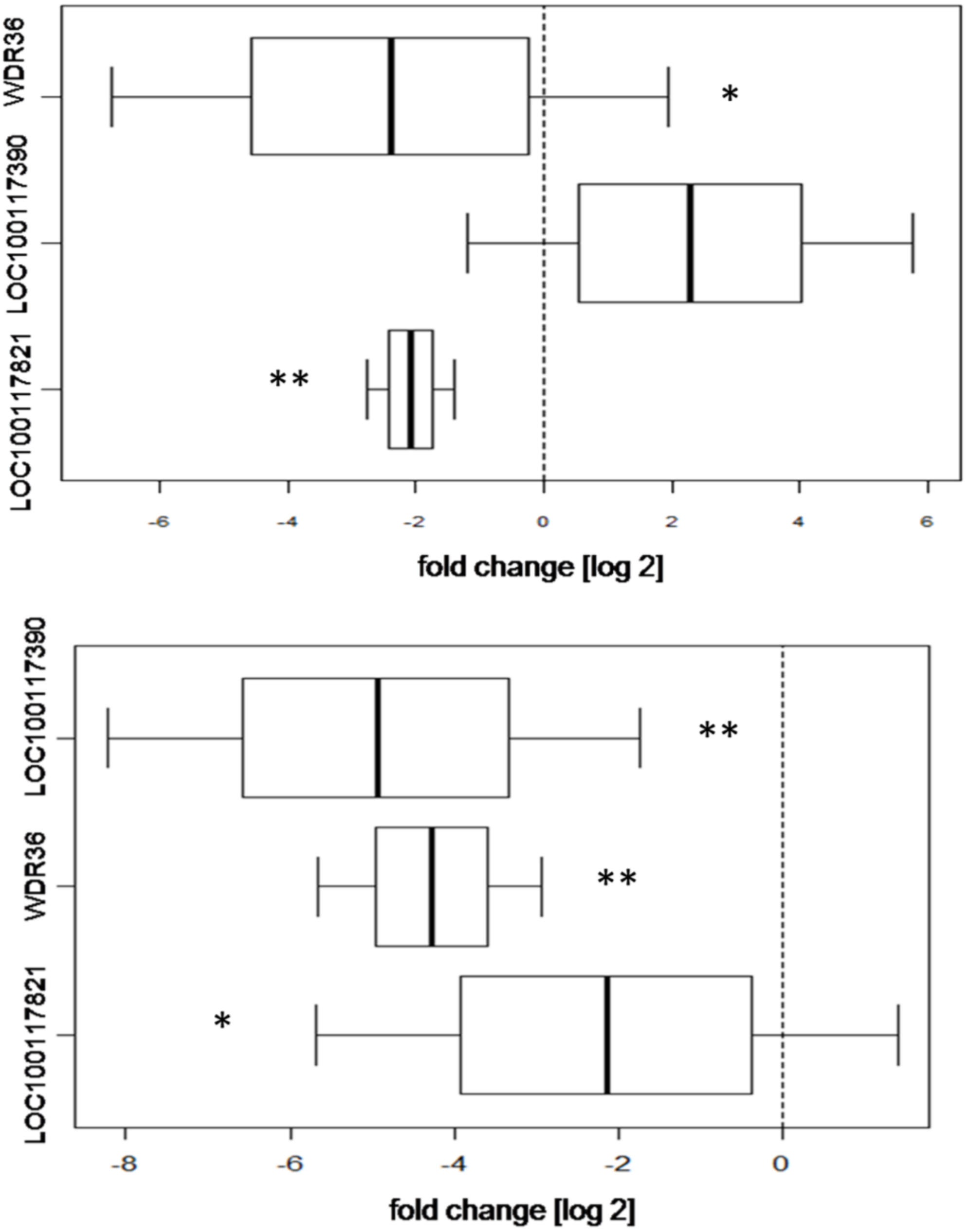

Supplement: Supplemental Material [file supp_gr.196204.115_Supplemental_lMaterial.pdf]
